# Psychometric properties of Indonesian slums dwellers’ place attachment

**DOI:** 10.1016/j.heliyon.2023.e19704

**Published:** 2023-09-06

**Authors:** Tery Setiawan, Missiliana Riasnugrahani, Edwin de Jong

**Affiliations:** a Department of Anthropology and Development Studies, Radboud University and Faculty of Psychology, Universitas Kristen Maranatha; b Faculty of Psychology, Universitas Kristen Maranatha; c Department of Anthropology and Development Studies, Radboud University

**Keywords:** Place attachment, Place identity, Slums, Social bonding, Indonesia

## Abstract

Social scientists have long considered place attachment to be an important factor in promoting environmentally sustainable behaviours among individuals. Raymond and colleagues have developed a five-factor place attachment measure, comprising place dependence, nature dependence, place attachment, family bonding, and friendship bonding, that encompasses most of the differentiations made and that has been amply tested for validity and reliability. However, the bulk of these confirmatory studies have been conducted in Western societies, neglecting people in the Global South and particularly people living in unstable, environmentally fragile regions such as slum areas. This study aims to fill this omission by testing the psychometric qualities of the five-factor place attachment measure in Indonesian slums using a dataset collected by the Resilient Indonesian Slums Envisioned (RISE) project. The dataset consists of a random sample of 700 respondents, living in slum areas of the cities of Bima, Manado, and Pontianak. We split the dataset into two and run factor analyses in EFA (*N* = 325) and CFA (*N* = 375) modes. Most notably, our results suggest a four-factor scale, in which place and nature dependences are merged into a single dimension. This finding seems logical considering that those living in urban slums are likely to have their natural surroundings, such as a river and its banks, as part of their living space. Overall, our study extends the use of place attachment to disaster-prone slum contexts that are often overlooked and, thus, supports the line of research that promotes environmental sustainability among people especially vulnerable to ecological changes.

## 1 Introduction

Discussions on person–place bonds started in the 1950s and, since then, this topic has received substantial attention from various disciplines [see 1,2]. Historically, these discussions revolved around the relation between human behavioural and environmental issues. Today, research in this field has extended to focus on cognitive functions, knowledge, and beliefs about aspects of the environment, emphasising individual subjective experiences and emotional attachment to the environment [1,2].

Therefore, the notion of place attachment is often used to refer to the connection of humans with their meaningful place. Put it simply, place attachment is an emotional tie between individuals and their place of living, identified by their identification with the place and their dependence on it [3,4]. Despite its establishment in the scholarly discussion, there seems to be varying conceptualizations and thus, operationalizations, of place attachment. Some scholars refer to a tripartite theoretical framework for getting to grips with place attachment [5]. The framework emphasises that people develop their attachment to a specific place through a *personal dimension*, such as religious and historical factors, through *place*, like social and physical factors, and through a *psychological process*, such as cognitive and affective evaluations. Along this line, Gustafson [6] offers another tripartite framework, in which he delineates the connections between the *self*, (e.g., activity, self-identification), *others* (e.g., social activities with others and community), and *environment* (e.g., physical distinctiveness).

Based on these conceptualizations, many have pursued various means for explaining place attachment. Some scholars lean more towards a unidimensional construct [7], such as measuring it through neighbourhood attachment [8]. While others measure it by using different dimensions, involving people, processes, and place [5]. Both unidimensional and multidimensional approaches, nevertheless, consider place as a living space where individuals are able to develop personal and social relationships, as well as attaining their means of living. Therefore, we can conclude that the notion of place attachment relies on two essential elements: (1) whether the place is psychologically distinctive and (2) whether the place allows individuals to realize their goals. In the words of Vaske and Cobrin [3], place attachment centres around place identity and place dependence. This, too, is reflected in the unidimensional approach, in which scholars claim that the components of identity, dependence, and attachment blend into one attitude [2]. Place identity is seen as an individual's feeling, or symbolic meaning, about the place that gives their life meaning and purpose, whereas place dependence is a functional connection to a place, reflecting how the physical setting supports the specific goals or desired use [9].

In the light of Vaske and Cobrin's work, Raymond et al. [10] argued that a more comprehensive framework is required to look at individuals' interactions with places in a natural or social context, and how these shape individuals' self-identity. They conceptualised place attachment as an individual's emotional or affective bond to a particular place and consider three contexts. First, the personal context that includes place identity and place dependence. Second, the community context that includes social bonding with family and peers. Finally, the natural environment context that covers natural bonding, affiliation, and connectedness with nature.

Based on the framework above, Raymond et al. [10] constructed a multidimensional scale to measure place attachment that included four dimensions: place identity, place dependence, social bonding, and nature bonding. The place identity and place dependence dimensions were based on a measure developed by Williams et al. [11]. The nature bonding dimension was developed based on descriptions of connectedness with nature from Kals et al. [12], and an additional item was added to this dimension based on the findings from semi-structured interviews with 30 local people in a southern Australian region [10]. Finally, the social bonding dimension was developed following measures proposed by Kyle et al. [13] and results from a thematic analysis of interviews conducted in the same region as the nature bonding measure discussed above. Their study reveals five dimensions of place attachment, in which the original social bonding dimension was split into friendship and family bonding [10].

In the extant literature, place attachment has been studied in relation to pro-environmental behaviour [14,15], environmentally responsible behaviour [16], and pro-tourism behavioural intentions, such as intention to revisit, positive word-of-mouth, and intention to recommend [[17], [18], [19], [20]]. In addition, previous studies have also found that emotional bonds to one's place of residence encourage people to engage in pro-environmental behaviour, such as protecting the place and assuming greater responsibility for the place's sustainability [21]. Other studies have extended the scope by focusing on how place attachment can even predict individuals' responses in coping with disasters [8], and how they behave in recreational parks [22]. Although these findings seem promising in times of rapid environmental change [23,24], Adewale et al. [25] argued that place attachment is too often studied under healthy environmental conditions, such as the availability of green open spaces, easy access to public services, low crime rates, and good job opportunities. These standards are assumed to be able to meet individuals' functional and emotional needs, and hence lead to a high level of place attachment.

In reality, however, many people do not enjoy such conditions. Slum areas, for instance, mostly lack these basic environmental and socioeconomic facilities. Kuffer et al. [26] note that an urban slum area may lack one or more of the following characteristics: 1) durable permanent housing that protects against extreme weather, 2) sufficient living space, 3) access to adequate clean water at an affordable cost, 4) access to adequate sanitation, and 5) security in preventing forced evictions. This is especially relevant in many developing countries with high rates of urbanization, such as India, China, Nigeria and Indonesia, which have contributed to a higher gap in supply and demand of decent settlements [25,27]. Consequently, we would expect such living conditions to hinder the development of people–place bonds [28].

Nevertheless, some studies have found that individuals living in slums can still feel attached to their neighbourhoods. For example, Li et al. [29] show that place attachment can enable people to survive and not want to move, even though an area is considered of poor physical and environmental quality. Other studies show that people who live in earthquake-prone or potential disaster areas are reluctant to evacuate [30], and even if they did, they would return to the area [8]. According to Li et al. [29], these individuals experience a dilemma between their high dependence on their social environment and their desire for a better life. This finding sheds new light on the importance of studying place attachment and its role in eliciting pro-environmental behaviours among people living in less-fortunate areas.

It follows that in this study we proclaim that such an investigation requires a valid measure that is relevant and has been validated among individuals living in environmentally disaster-prone and socially and economically challenging areas, such as slums. The reason is two-fold. First, slums often do not offer the luxury of good and clean environmental surroundings [31,32]. Second, people living in these areas are often forced to stay due to the unintended consequences of urbanization as mentioned in the earlier literature [25,27]. In line with this, Indonesia provides an appropriate setting for the study of slum areas. Considered as the most disaster-prone country in the world [33], Indonesia's development hinges on the fact that there are still a large number of slum-dwellers spread across the country [34]. This combination puts the slum dwellers at a much greater risk of ecological change than many others. In order to respond, it is vital to test the validity of place attachment among people living in Indonesian slums to enable politicians and policymakers to react in appropriate ways. Nevertheless, such studies are scarce in the current literature.

In order to fill this gap, we test the psychometric qualities of Raymond et al.'s [10] place attachment measure in Indonesian slums. The scale consists of five dimensions, and we opted for this version as it distinguishes between family and friendship bonding, which seemed to be reflected in the areas under study. The scale is tested using a dataset of the “Resilient Indonesian Slums Envisioned (RISE)” project that was collected in 2021 and which focused on the water management and relational wellbeing of people living in slums in the three Indonesian cities of Bima, Manado, and Pontianak. These cities reflect most of the social and water challenges that Indonesian cities are nowadays facing and were selected after careful consideration of the latest report on cities with high disaster risks in a study by the World Bank [27,35].

## 2 Method

This study forms part of the larger RISE project that was launched in the autumn of 2021. The project aims to map social-ecological interactions with the goal of making Indonesian cities more resilient to water-related disasters, while also considering other relevant factors such as place attachment. The survey documentation is publicly available online through online archiving system. In this paper we briefly outline the sampling method and, for a fuller explanation, readers are directed towards the survey documentation.

### 2.1 Selection of locations

For this study, we purposively selected slum areas as our research locations. There are a substantial number of slum areas across Indonesia, and we purposively based our selection of locations on two criteria. First, since this study concerns ecological change that may impoverish the living conditions of people living in slums, we focused on areas where there has been an increased risk of flooding as documented by the latest World Bank report [27]. We further narrowed down the selection to cities that have experienced recent major floods while often being overlooked. According to the World Bank report, of the many Indonesian cities, Bima, Manado, and Pontianak face heightened flood risks [36]. This circumstance has in part been exacerbated by the increased economic activity that has pushed settlements in these cities to expand to riskier areas along the rivers and coastal areas while often accompanied by poor flood mitigation standards. Second, to ensure that the current study is relevant to the national and local governments, the focal slum areas in each city should be in line with the categorisation of slums (*kawasan kumuh* in Indonesian) governed by Law No. 1 of 2011 on Housing and Residential Areas of Indonesia. As a result, the selection of slum areas also corresponds to the mayoral decrees in the corresponding cities (Mayor's decree of Pontianak Number 1063.1/D-PRKP/Year 2020; Mayor's decree of Bima No. 188.45/747/650/XII/2019; and Mayor's decree of Manado No. 163 of 2015).

More specifically, the selected locations of slums are the following: 1) the subdistricts of Paruga and Sarae in Bima, 2) the subdistricts of North Titiwungen and Wawonasa in Manado, and 3) the subdistricts of Tambelan Sampit, Sungai Jawi Luar, and Tengah in Pontianak. Based on rapid appraisals, these areas have at least two of the following slum criteria: 1) lack of access to clean water and a proper sanitation system, 2) frequent fluvial and/or pluvial floods either in the immediate area or in the surrounding main streets, and 3) poor sewage system.

### 2.2 Random selection of households and respondents

The data collection process in the three cities started in November 2021 and lasted until February 2022. The permit had been granted by The Directorate General of Politics and General Administration from The Ministry of Home Affairs of the Republic of Indonesia (470.02/7428/Polpum) and by the Research Ethical Committee of Universitas Kristen Maranatha (No.134/KEP/X/2022). The study aimed to gather a random sample from the general population aged 18 and above in each location. To this end, the study employed a random walk method with a sampling criterion of having lived in the area for at least three years. The data collection began the random walks by selecting a starting point or house that was near to the local government office in each sub-district. Subsequently, the survey used two-house intervals to move to the next household until it achieved the targeted sample size. The study aimed to acquire 300 respondents from each city and hence a total of 900 participants from three cities.

Within each household where there was more than one adult eligible to participate, the surveyor picked the adult whose birthday was closest to the day of the survey. Next, prior to their participation, the surveyor informed the identified respondent about the study, and asked them for their informed consent to voluntarily participate in the study, while making clear they could refuse. Those who agreed were asked for their written consent. After the study, each participant received Rp. 50.000,00 (approximately €3) as a token of appreciation.

The survey successfully collected information from 700 of the 920 respondents approached. In total, there were 262 males and 438 females spread across the three cities. In more detail, there were 300 respondents from Pontianak (150 males and 150 females), 200 respondents from Bima (46 males and 154 females), and 200 respondents from Manado (66 males and 134 females). Their average age was 43 (*SD* = 11.79).

### 2.3 Measures

There are several measures of place attachment and we considered the five-factor scale by Raymond et al. (2010) to be the most appropriate given our research context for two reasons. First, place attachment should be considered as part of an individual's identity. Similar to the notion of individual attachment that develops over time, attachment to a particular place to some extent reflects the centrality of that place to the individual [37]. Childhood experience plays a major role in determining whether individuals develop a strong attachment to a place. Logically, the longer individuals stay in a specific place, the stronger the attachment to that place becomes. A place can be seen as the field where all individual experiences occur, socially, personally, as well as environmentally, which fits well with the tripartite concept in Raymond et al.’s measurement model. Second, the measurement accommodates the two distinct categorisations of social connection, namely family and friend bonding. This, we felt, reflected the reality of our research context where family connections are a strong determinant in making people stay and not wanting to move away. Further, slums generally expand due to economic migration and some people move in without having a family connection but instead through the persuasion of their friends [38]. Therefore, by distinguishing the two social categories, the measure enables us to investigate the type of connection that individuals have.

The original scale consists of 19 items spread across 5 dimensions. For the purpose of this research, we added several further items, two items for place identity, one item for place dependence, and one item each for the family and the friend bonding dimensions. In total, the measure therefore consists of 24 items spread across the five dimensions (see Appendix 1 for a full list). The place identity dimension asks respondents to rate themselves on statements such as “I am very attached to (name of place)”. The nature bonding dimension is represented by statements such as “I feel one with the natural environment when I spend my time in the natural environment at (name of place)”. Meanwhile, the place dependence dimension asks respondents to rate themselves on statements such as “I learn a lot about myself when I spend my time in nature at (name of place)”. Finally, the family and friend bonding dimensions ask respondents to rate themselves on statements such as “Without my family in (name of place) I might move out” and “The friendships formed through sports activities in (name of place) are very important to me”, respectively. All the statements were rated on six-point Likert scales, ranging from 1 strongly disagree to 6 strongly agree.

The original English scale was translated to Indonesian language using a back-translation method [39]. In detail, firstly, the initial translation involved five Indonesian scholars from various disciplines, such as psychology, economics, anthropology and development studies. This panel of experts translated the original scale to the Indonesian language. Secondly, the research team of the RISE project, back-translated the items to the source language. Finally, along with the panel and the addition of two people from the target population, the research team held several rounds of verbal discussion to determine the translation validation. The discussions centred around the topics of comparability of language and the similarity of interpretation of the two versions. By combining a panel of experts and the target population, the translation validation process aimed to achieve a fine balance between formal translation of the scale and a high level of readability.

### 2.4 Analysis strategy

In order to rigorously test the psychometric qualities of the measure, we employed exploratory factor analysis (EFA) which was followed by confirmatory factor analysis (CFA). EFA is suitable for researchers who want to find out if one or more latent variables are related to the manifest variables [40]. This is done by partitioning a shared variance from a variable from its unique and error variances. CFA, on the other hand, is suitable to confirm an existing factor-variable (or factor-item) configuration [41]. In doing so, we adopted the following strategy. First, we randomly split the dataset into two in SPSS to enable us to run an exploratory factor analysis (EFA) followed by a confirmatory factor analysis (CFA) of the measures.

To do this, we used the original dataset containing all 700 cases and then went to “Select Cases” option in SPSS and specified approximately 50% of the cases. We chose the option “Filter out unselected cases” to create a filter variable (SPSS automatically provides 1 = selected cases and 0 = unselected cases). After this, we created two new dataset files that have been randomly split and coded by the filter variable (the full syntax is shown in Appendix 4). As a result, we acquired *N =*325 for EFA and *N =*375 for CFA. As a rule of thumb in determining an adequate sample size, we followed Costello and Osborne's study [40], in which they found that 62.9% of 303 PscyhINFO (studied in 2005) used a subject to item ratio of 10:1. As for CFA, Kyriazos [41] concluded that a sample size of *N* ≥ 200 is considered an adequate size. Table 1 provides more details of the individual characteristics of respondents based on analysis division.

**Table 1:** Respondent characteristics based on the different types of factor analyses.

Predictors	Range	Bima		Pontianak		Manado		*F test*
		M	SD	M	SD	M	SD	
Individual characteristics among EFA sample (*N* = 325)								
Age	–	46.12	12.38	40.02	11.12	44.01	12.09	*F*(2,322) = **8.19**
Gender (female as reference)	0–1	.23	.42	.55	.50	.31	.47	
Educational level	1–10	4.05	1.91	3.91	1.84	3.65	1.36	*F*(2,322) = 1.26
Individual income	1–10	2.34	1.64	3.84	2.12	8.47	19.70	*F*(2,322) = **8.79**
**Individual characteristics among CFA sample (*N*** = 3**75)**								
Age	–	45.00	13.05	41.70	10.16	43.27	11.96	*F*(2,372) = 2.58
Gender (female as reference)	0–1	.22	.42	.45	.50	.34	.48	
Educational level	1–10	4.18	1.62	3.57	1.66	3.76	1.13	*F*(2,322) = **5.23**
Individual income	1–10	2.29	1.73	3.74	2.10	7.60	17.71	*F*(2,322) = **8.71**

Note*:* Bold indicates significance at the *p<.05* level.

Before we proceed to lay out several reasons why it was necessary to perform a consecutive EFA and CFA techniques in the scale validation, it is worth reiterating the main objective of this study: Although place attachment has been shown to relate to pro-environmental behaviours in many different settings, e.g., rural areas, tourism setting, the extant literature lacks studies on areas where there are lacks of physical infrastructures, e.g., slum settlements. Due to a completely different physical setting from previous studies, we expect that there will be distinct ways of perceiving place of residence among our samples. Thus, EFA and CFA were required to serve the aim of the study.

Specifically, the reasons are as follows: One, EFA was useful to loosely test whether the measure would indeed result in five factors as the theoretical claims, while CFA confirms the identified factor-item configurations [42]. Two, EFA was necessary to identify potential measurement problems in the dataset, such as low factor loadings, whereas CFA was used to test and modify, if necessary, the identified measurement model. Finally, EFA was useful to layout a common factor model, while CFA was necessary to test the goodness-of-fit of the identified model [43,44].

For the EFA, we employed the following parameters: First, we used the estimation method of common factor model in form of iterative principal axis factoring (PAF) in SPSS 27 [42]. This is mainly because PAF considers measurement error, unlike principal component analysis (PCA), and it does not require multivariate normality. Therefore, employing PAF method allows us to loosely map out the factor-item configuration but still maintains the appropriate level of values of variance accounted for by the identified factors. Second, we opted for oblique rotation, specifically Oblimin rotation, because theoretically the factors proposed by Raymond et al. [10] are thought to be correlated. In analysing the results, it is possible to switch to a different type of rotation, such as Promax rotation, depending on the initial results. Third, the number of factors retained would initially follow the theoretical claim, that is five factors. However, we deemed a factor to be stable if its eigenvalue was at least 1 [40]. Therefore, although the established scale and its theory provide us with strong theoretical base, we would still make necessary changes to follow the statistical findings during the validation process. Finally, fifth, to ensure that the variables (items) were strongly correlated with their corresponding factors, we set a minimum value for communality at 0.4 [45]. Item configurations can be modified, such as by removing certain items, if there is a low factor loading or a double loading. Although many set the minimum acceptable factor loading at 0.3, we followed Peterson [46] in setting our threshold at a minimum of 0.4.

Next, the CFA, by comparing the results with the EFA model, was used to confirm whether the data fitted the theory. We used maximum likelihood (ML) as the estimation method. By using ML, we were able to confirm that there are relationships between factors, the configuration of the indicators or items being measured, and how they relate to the factor loadings [42]. To this end, we used the lavaan package in R [47]. By default, the measurement of the first item of a factor is set to a value of 1 and thus, determining the scale of the factor. Additionally, there is an automatic inclusion of residual variances. Lastly, there is an assumption of correlation between all the factors considered as independent variables. In setting criteria, first, we complied with the good-fit guidelines proposed by Lance et al. [48] and Hooper et al. [44]. In other words, the comparative fit index (CFI) should be greater than 0.90 [48], the root mean square error of approximation (RMSEA) should be less than 0.07 [49], and the standardised root mean squared residual (SRMR) should be less than 0.08.

Second, the average variance extracted (AVE) and composite reliability (CR) would be used to assess the factors' convergent validity [50]. The AVE value should be at least 0.5 and the CR a minimum of 0.6. However, Fornell and Larcker [51] argue that even if the AVE is below 0.5, provided but the CR is above 0.6, the factor can still be considered valid. Moreover, the AVE value should be larger than the shared variance with other constructs, determined by the factor correlations, to ensure the factors’ discriminant validity [52]. Finally, the reliability level of each factor should be at least 0.6 [53]. We applied all these criteria in determining the psychometric properties of a modified place attachment measure, based on Raymond et al. [10], when applied to people living in slums in Indonesia.

## 3 Results

We start by providing the resulting factor structures from the EFA and CFA. Following this, we discuss the item-factor configuration and the internal consistency of all the factors. Finally, we conclude by discussing the convergent and discriminant validity of the factors.

### 3.1 Factor structure

Initially, we ran EFA using principal axis factoring (PAF) given our expectation of non-orthogonal factors. The anticipation of non-orthogonal factors is mainly due to the tripartite model proposed by the measurement theory [10,13]. Considering a place to be a field of individual experiences allows the notion of a place to also become a source of connection to social life and nature [10]. Thus, all the factors included in the measure should be considered as having a shared variance. Here, we started with Oblimin rotation and set the eigenvalues to 1. Later after the initial results, we switched to a Promax rotation after the removal of several items and the merging of place and nature dependence factors.

From the results, we focussed at the sampling adequacy through the Kaiser Meyer Olkin (KMO) and Bartlett's sphericity test, the factor configuration, and the correlations between factors (see Appendix 2 for more details). KMO is used to measure the degree of sampling adequacy of the dataset and Bartlett's sphericity test is used to check whether the data produced is not identical to the original correlation matrix [54,55]. The sampling adequacy of the model is considered high when the value of KMO is at least 0.90 and the Bartlett's sphericity test's significance value is less than 0.05 [54]; and all our models meet this threshold. This suggests that our sample is sufficient and our data does not produce an identity matrix similar to the original correlation matrix. As such, the data appeared to be acceptable for further analysis.

Our next finding is that rather than the assumed five-factor model, the analysis produces stable four-factor models indicated by Eigenvalues above 1. The difference between the tested models is in the number of items included. In the first model (Model 1 in Appendix 2), although the factor structure appears acceptable, the pattern matrix reveals some items (items 8, 16, 17, and 18) that load onto multiple factors. After removing these items, one by one, we arrive at the final model shown in Table 2 (Model 3 in Appendix 2) which shows a sufficient level of sampling adequacy, Eigenvalue, and the Cronbach's Alpha.

**Table 2:** Goodness of fit for EFA and CFA.

Parameters	EFA				CFA			
	F1	F2	F3	F4	F1	F2	F3	F4
KMO and Bartlett's sphericity test	.90 (*p = .*00)							
Eigenvalue	9.90	2.51	1.74	1.22				
Variance explained (%)	48.13	11.13	7.19	4.82				
χ2					823.44 (154, p = .00)			
CFI					.91			
RMSEA					.11			
SRMR					.05			
CR					.94	.82	.74	.71
AVE					.68	.40	.49	.46
Cronbach's Alpha	.95	.93	.83	.79	.95	.95	.83	.78

Next, the CFA was run according to the identified factor-item configurations from the final model of the EFA (see Appendix 3 for full detail). Table 2 shows the goodness of fit of the final CFA model. The first two CFA models that we ran do not fit the data. We used modification indices (MIs) to evaluate the statistical significance of various unspecified relationships between items [56]. It is claimed that MIs greater than 3.84 are considered statistically significant (p < .05), and by freeing such parameter would significantly improve model fit. However, we would apply MIs starting with the largest unspecified relationships. The MIs calculated using the lavaan package show that there are items that are correlated which could potentially improve the model fit, e.g., item 11 is correlated with item 10 [47]. We repeat this procedure until we reach at the final model shown in Table 2 (Model 3 in Appendix 3) which is a better fit to the data (for full description of MIs, please refer to the syntax in Appendix 4). Although the chi-square test shows a significant discrepancy between ‘the sample and fitted correlation matrix’ [52, p.2], many scholars argue that this test is highly sensitive to sample size [e.g., 46]. As such, it is likely that a model based on a larger sample size would be significant. In the final model, the values of CFI (0.91) and SRMR (0.05) are within the acceptable range, but the RMSEA is outside the suggested range (≤0.10) [48,49]. However, Hu and Bentler [52, p.27] suggest that a value of CFI close to 0.95 in combination with a value of SRMR close to 0.09 is sufficient to conclude there is a good model fit. Therefore, we conclude that the final model is an appropriately specified model for our data.

### 3.2 Item-factor configuration (factor loadings)

Having concluded this, we turn our attention to the factor scores (or loadings) of the scale. Table 3, Table 4 show the factor loadings of all the items in the final model. From the EFA results (see Table 3), we can conclude that the communality of all the items are in the medium to high range [40]. This means that a good proportion of the variance is explained by each item. The two factors, place dependence and nature dependence, that are viewed as distinct by Raymond et al. [10], are shown to converge into a single factor. Looking at each item, we observe a well configured item positioned in its corresponding factor. For each factor, the loadings are in the medium to high range. This judgement is supported by the high CR and a good AVE of the CFA model (see Table 4). Although some factors are found to have an AVE below the suggested value [50], the CRs of those factors are high. On this basis, the factors are considered to demonstrate an acceptable level of convergent validity [see 53]. In addition, the AVE values of all the factors exceed the correlation coefficient between each factor and all the other factors. As such, each factor shows a good level of discriminant validity [57]. Finally, each factor is also found to be highly reliable based on the high Cronbach's alphas [53].

**Table 3:** The EFA factor loadings of the final model.

Construct					
Place attachment	*h*^*2*^	Factor loading			
		F1 Place & nature dependence	F2 Place identity	F3 Family bonding	F4 Friend bonding
11. I learn a lot about myself when I spend my time in the natural environment at (name of place)	.84	.93			
12. I feel very attached to the natural environment at (name of place)	.81	.92			
13. I feel at peace when I spend my time in the natural environment at (name of place)	.80	.89			
9. I feel one with the natural environment when I spend my time in the natural environment at (name of place)	.80	.87			
10. My bond with (name of place) will diminish if the plants and animals that live in (name of place) disappear	.77	.84			
15. (Name of place) is incomparable	.71	.69			
14. I feel more satisfied living in (name of place) than any other place	.63	.64			
3. I'm very attached to (name of place)	.81		.90		
2. (name of place) is important to me	.70		.83		
1. This place means a lot to me	.68		.81		
4. I have a lot of fond memories of (name of place)	.73		.80		
6. I feel (name of place) is a part of me	.71		.75		
5. I strongly identify myself with (name of place)	.70		.74		
7. I feel comfortable living in (name of place)	.56		.73		
19. I live in (name of place) because my family also lives here	.84			.95	
20. The relationship with my family in (name of place) is very special to me	.83			.92	
21. Without my family in (name of place) I might move out	.50			.50	
23. The friendships formed through sports activities in (name of place) are very important to me	.51				.89
24. Without my old friends in (name of place) I might move out	.72				.76
22. The friendships formed through communal activities in (name of place) are very important to me	.64				.52

**Table 4:** The CFA factor loadings of the final model.

Construct Place attachment	Unstandardised factor loading (standard errors)			
	F1 Place & nature dependence	F2 Place identity	F3 Family bonding	F4 Friend bonding
11. I learn a lot about myself when I spend my time in the natural environment at (name of place)	.93			
12. I feel very attached to the natural environment at (name of place)	.80 (.03)			
13. I feel at peace when I spend my time in the natural environment at (name of place)	.85 (.03)			
9. I feel one with the natural environment when I spend my time in the natural environment at (name of place)	.87 (.04)			
10. My bond with (name of place) will diminish if the plants and animals that live in (name of place) disappear	.86 (.03)			
15. (Name of place) is incomparable	.74 (.03)			
14. I feel more satisfied living in (name of place) than any other place	.71 (.03)			
3. I'm very attached to (name of place)		.69		
2. (name of place) is important to me		.57 (.04)		
1. This place means a lot to me		.60 (.04)		
4. I have a lot of fond memories of (name of place)		.67 (.04)		
6. I feel (name of place) is a part of me		.60 (.04)		
5. I strongly identify myself with (name of place)		.70 (.03)		
7. I feel comfortable living in (name of place)		.58 (.04)		
19. I live in (name of place) because my family also lives here			.80	
20. The relationship with my family in (name of place) is very special to me			.64 (.04)	
21. Without my family in (name of place) I might move out			.66 (.06)	
23. The friendships formed through sports activities in (name of place) are very important to me				.68
24. Without my old friends in (name of place) I might move out				.74 (.09)
22. The friendships formed through communal activities in (name of place) are very important to me				.60 (.06)

Overall, this evidence of robust psychometric properties holds for alternative models using the full dataset, *N* = 700. That is, this claim is supported by both the 10.13039/100007411EFA and CFA statistical techniques.

## 4 Discussion and conclusions

The purpose of this study has been to examine the psychometric qualities of the place attachment measure proposed by Raymond et al. [10] when applied in Indonesian slums. Our findings show that, after some modification, the measure is psychometrically valid for a specific sample of people living in slums. We start the discussion by explaining the findings related to the latent constructs of the measure and continue with their indicators (items).

First and foremost, our findings support the consensus notion of place attachment, defined as an emotional tie between an individual and a particular place, along with the people in it [37,58]. This definition fits with sociological and psychological perspectives, in which an attachment to place cannot be understood separately from an individual's attachment to people living in that place. Here, our psychometric findings demonstrate that, indeed, a place attachment measure should, in the very least, consist of place dependence and place identity [3]. Furthermore, compared to the original five-factor scale by Raymond et al. [10], we find that our data fit better with a four-factor scale. The four factors are place and nature dependence, place identity, family bonding, and friend bonding. This finding, which combines the place dependence and nature dependence of the earlier model, is not surprising given the research context. That is, place and nature dependence should be viewed as a single latent construct because, for those living in slums or other ‘less-accessible’ areas, their area of residence is likely to include natural surroundings such as mountains and rivers and, when they do, this nature merges into their living space. This finding echoes claims made in previous studies by Fedele et al. [23] and by Williams and Vaske [9], in which place dependence significantly overlaps with nature attachment. In effect, nature, as a place, provides individuals with basic needs such as water, construction material, and even recreational opportunities [23]. This is very apparent to our population groups who have limited living space and where nature and their home coincide in one dimension of place. Therefore, we conclude that we have a valid measure of place attachment that is relevant within the context of people living in less-fortunate areas.

Second, given the merging of place dependence and nature-bonding factors, we should look at the item–factor configuration. Our findings show that the items in the original factors fit nicely in the new combined factor. Items such as “I feel very attached to the natural environment at (name of place)”, which originally represented the nature-bonding factor, and “I feel more satisfied living in (name of place) than any other place”, as an item of place dependence, fit well in new factor of place and nature dependence. Theoretically, this fits with the claim by Vaske and Kobrin [3] that place dependence is a functional attachment through which people feel dependent upon their place due to the amenities it provides such as hiking opportunities and feeling at one with nature. As such, those who are attached to the natural environment of their place of residence are likely to be satisfied, with and dependent on, that place. Methodologically, the items are shown to share a substantial variance indicating a strong association between items.

Third, for the other factors, i.e. place identity, family bonding, and friend bonding, we show that the items provided by Raymond et al. [10] plus our additional items (e.g. “Without my old friends in (name of place), I might move out”), provide a valid and reliable measure of their corresponding factors. The factors are shown to be stable after multiple runs of the model and display a good reliability. Moreover, there are marked differences in the levels of importance (i.e. total variance explained) of all the four factors when compared to the scale of Raymond et al. [10]. They found that the most important factor is place identity followed by nature bonding. In our four-factor model, the most influential factor is place and nature dependence, with place identity in second place. There are various possible explanations for this difference, but we believe the reason is as follows. First, due to the dissimilar type of respondents in our study, one should be open to the possibility that the factor configuration is different. Given that our respondents are living in slum areas with little or no option to improve their living space, it is not that surprising that they perceive a high dependence on their place and the natural environment that comes with it [59]. In addition, having merged the place dependence and nature bonding factors, this more-encompassing factor is likely to have a greater importance and explain more of the variance.

### 4.1 Main implications

Overall, the main implications of this research are as follows. First, the concept of place attachment is also applicable for slum areas. Although slum dwellers do not live in good or healthy environmental conditions, they can still develop an emotional tie to their place of residence. This result confirms that place attachment is largely a subjective evaluation of individuals' place of residence, which involves a sense of emotional attachment not only to their living space, but also to their community, and their identity related to the place. More importantly, our findings confirm that place attachment hinges on whether the living space is able to fulfil individuals’ needs, e.g., basic needs, and to develop their social identity. This research supports previous findings in China [29] that even though people may live in a poor-quality environment, they do not want to leave their locations.

Second, place attachment in slum areas has a unique factor that combines the dependence on place and nature. We argue that this is highly relevant considering the characteristics of most slum areas. In the cities we studied, all the slum areas are surrounded by natural environments, like rivers or lakes. However, due to dense populations these resources are often used for daily living, such as for water sources and optimizing riverbanks for settlements. This is mostly due to slum characteristics of lacking public facilities, and the absence of capital and labour. Therefore, nature dependency cannot be viewed as solely a natural recreation as in previous measures. Slum dwellers are rather highly dependent on natural resources and ecosystem services to fulfil their daily lives [60]. Consequently, we can take advantage of this notion in further research by using community knowledge of local land, forests, and water resources, to prevent environmental damage.

Third, our findings indicate that place and nature dependency is the strongest factor in relation to place attachment in slum areas. This finding is of importance when scholars aim for a quick snapshot of place attachment by simply focusing on this sole factor. Of course, in order to deliver a high quality study scholars are encouraged to conduct a principal component analysis (PCA) prior to testing.

### 4.2 Limitations

We recognise that our study has some limitations. First, although we have successfully engaged with a sizable sample from three different slum areas in Indonesia, one should not take the generalisability of the measure for granted. For instance, the findings might be different if we repeated our study in Jakarta, where the slums are notorious for being controlled by slum landlords [31]. This could result in a different picture of place attachment because the individuals’ presence there may be largely driven by economic motives. Second, our study does not include other relevant scales that could be correlated with the place attachment measure in an attempt to show the predictive validity of the measure. Previous studies have argued that place attachment is strongly linked with environmentally-responsible behaviour and risk perception [3,61,62]. Thus, a follow-up study could usefully take other measures into account in an attempt to explain the predictive function of place attachment.

In conclusion, we show that our modified place attachment measure, inspired by Raymond et al. [10], is a valid measure for investigating place attachment among people living in slums and probably in other areas where access to basics is scarce. Our findings show that the measure has good psychometric properties and consists of four factors: place and nature dependence, place identity, family bonding, and friend bonding. The modification to the five-factor model does not amount to major differences with the previously found psychometric properties of existing measures [10,3,13]. The two main factors of place dependence and place identity in the earlier measures are still present in our model. While there has been some overlap in the terms, such as social bonding with family and friend bonding with other measures, our measure still includes the conceptualisation of place attachment. Our findings also greatly reflect the lives of many Indonesians living in urban areas, where the conception of nature is embedded in their living space due to their limited access to open natural fields or natural parks [63]. Despite the limitations outlined above, our findings are useful in providing psychometric evidence of a place attachment measure that is relevant for areas similar to our research context. As such, our study is useful in extending the use of place attachment to disaster-prone slum contexts that have often been overlooked.

## Author contribution statement

Tery Setiawan; Missiliana Riasnugrahani; Edwin de Jong: Conceived and designed the experiments; Performed the experiments; Analyzed and interpreted the data; Contributed reagents, materials, analysis tools or data; Wrote the paper.

## Data availability statement

Data associated with this study has been deposited at DANS EASY platform: https://doi.org/10.17026/dans-z5q-d3ae.

## Declaration of competing interest

The authors declare that they have no known competing financial interests or personal relationships that could have appeared to influence the work reported in this paper.

## Figures and Tables

**Appendix 1:** The original scale of place attachment handed to respondents (*N items = 24*)

Measure	
Place attachment	Dimension
1. This place means a lot to me	Place identity
2. (name of place) is important to me	Place identity
3. I'm very attached to (name of place)	Place identity
4. I have a lot of fond memories about (name of place)	Place identity
5. I identify strongly myself with (name of place)	Place identity
6. I feel (name of place) is a part of me	Place identity
7. I feel comfortable living in (name of place) (*additional item*)	Place identity
8. I feel happy to be back again to (name of place) after a having been gone a long time (*additional item*)	Place identity
9. I feel one with the natural environment when I spend my time with the natural environment at (name of place)	Nature bonding
10. My bond with (name of place) will diminish if the plants and animals that live in (name of place) disappear	Nature bonding
11. I learn a lot about myself when I spend my time in the natural environment at (name of place)	Nature bonding
12. I feel very attached to the natural environment at (name of place)	Nature bonding
13. I feel peace when I spend my time in the natural environment at (name of place)	Nature bonding
14. I feel more satisfied living in (name of place) than any other place	Place dependence
15. (Name of place) is incomparable	Place dependence
16. I will not replace with any other place for activities that I do in (name of place)	Place dependence
17. For me, doing activities in (name of place) is more important than doing them in any other place	Place dependence
18. (Name of place) is the best place to do activities that I like (*additional item*)	Place dependence
19. I live in (name of place) because my family also lives in here	Family bonding
20. The relationship with my family in (name of place) is very special to me	Family bonding
21. Without my family in (name of place) I might move out (*additional item*)	Family bonding
22. The friendships formed through communal activities in (name of place) are very important to me	Friend bonding
23. The friendships formed through sports activities in (name of place) are very important to me	Friend bonding
24. Without my old friends in (name of place) I might move out (*additional item*)	Friend bonding

**Appendix 2:** Correlations between factors and goodness of fit of the EFA models

Factors	Model 1				Model 2				Model 3			
	F1	F2	F3	F4	F1	F2	F3	F4	F1	F2	F3	F4
F1	–	**.21**	**−.59**	**.35**	–	**.19**	**−.62**	**.34**	–	**.65**	**.24**	**.40**
F2	–	–	**−.31**	**.36**	–	–	**−.37**	**.37**	–	–	**.43**	**.43**
F3	–	–	–	**−.35**	–	–	–	**−.34**	–	–	–	**.45**
KMO and Bartlett's sphericity test	.92 (*p* = .00)				.91 (*p* = .00)				.90 (*p* = .00)			
Eigenvalue	12.30	2.53	1.97	1.35	10.52	2.23	1.80	1.24	9.90	2.51	1.74	1.22
Variance explained	50.12	9.35	7.03	4.40	48.79	10.64	7.14	4.65	48.13	11.13	7.19	4.82

Note*:* Figures in bold indicate significance at the *p<.05* level. **F1:** Place & nature dependence; **F2:** Place identity; **F3:** Family bonding; **F4:** Friend bonding.

**Appendix 3:** Correlations between factors and goodness of fit of the CFA models

Factors	Model 1				Model 2				Model 3			
	F1	F2	F3	F4	F1	F2	F3	F4	F1	F2	F3	F4
F1	–	**.31**	**.43**	**.38**	–	**.31**	**.42**	**.37**	–	**.32**	**.42**	**.38**
F2	–	–	**.26**	**.27**	–	–	**.26**	**.27**	–	–	**.26**	**.27**
F3	–	–	–	**.27**	–	–	–	**.27**	–	–	–	**.26**
χ2	1108.45 (164, *p* = .00)				.992 (160, *p* = .00)				823.44 (154*, p* = .00)			
CFI	.87				.89				.91			
RMSEA	.12				.12				.11			
SRMR	.06				.06				.05			

## Appendix 4 The full syntax of EFA and CFA operations

# splitting dataset in SPSS 27 #

USE ALL.

COMPUTE filter_$=(uniform(1) ≤ 0.50).

VARIABLE LABELS filter_$ 'Approximately 50% of the cases (SAMPLE)'.

FORMATS filter_$ (f1.0).

FILTER BY filter_$.

EXECUTE.

#creating a dataset for selected cases(1) #

DATASET COPY data2.

DATASET ACTIVATE data2.

FILTER OFF.

USE ALL.

SELECT IF (NOT(filter_$ = 0)).

EXECUTE.

DATASET ACTIVATE DataSet1.

# creating a dataset for the remaining cases(0) #

DATASET COPY data3.

DATASET ACTIVATE data3.

FILTER OFF.

USE ALL.

SELECT IF (filter_$ = 0).

EXECUTE.

DATASET ACTIVATE DataSet1.

#PAF (EFA) syntax using the first split dataset (data2) #

# 1st model.

FACTOR.

/VARIABLES PA_PI1 PA2 PA3 PA4 PA5 PA6 PA7 PA8 PA9 PA10 PA11 PA12.

PA13 PA14 PA15 PA16 PA17 PA18 PA19 PA20 PA21 PA22 PA23 PA24.

/MISSING LISTWISE.

/ANALYSIS PA_PI1 PA2 PA3 PA4 PA5 PA6 PA7 PA8 PA9 PA10 PA11 PA12.

PA13 PA14 PA15 PA16 PA17 PA18 PA19 PA20 PA21 PA22 PA23 PA24.

/PRINT UNIVARIATE INITIAL KMO EXTRACTION ROTATION.

/FORMAT SORT BLANK(0.30)

/CRITERIA MINEIGEN(1) ITERATE(25)

/EXTRACTION PAF.

/CRITERIA ITERATE(25)

/ROTATION OBLIMIN.

/METHOD = CORRELATION.

# 2nd model remove PA16 PA17 PA8 due to double loadings.

FACTOR.

/VARIABLES PA_PI1 PA2 PA3 PA4 PA5 PA6 PA7 PA8 PA9 PA10 PA11 PA12.

PA13 PA14 PA15 PA16 PA17 PA18 PA19 PA20 PA21 PA22 PA23 PA24.

/MISSING LISTWISE.

/ANALYSIS PA_PI1 PA2 PA3 PA4 PA5 PA6 PA7 PA9 PA10 PA11 PA12.

PA13 PA14 PA15 PA18 PA19 PA20 PA21 PA22 PA23 PA24.

/PRINT UNIVARIATE INITIAL KMO EXTRACTION ROTATION.

/FORMAT SORT BLANK(0.30)

/CRITERIA FACTORS(4) ITERATE(25)

/EXTRACTION PAF.

/CRITERIA ITERATE(25)

/ROTATION OBLIMIN.

/METHOD = CORRELATION.

# 3rd model remove PA18 due to double loadings.

FACTOR.

/VARIABLES PA_PI1 PA2 PA3 PA4 PA5 PA6 PA7 PA8 PA9 PA10 PA11 PA12.

PA13 PA14 PA15 PA16 PA17 PA18 PA19 PA20 PA21 PA22 PA23 PA24.

/MISSING LISTWISE.

/ANALYSIS PA_PI1 PA2 PA3 PA4 PA5 PA6 PA7 PA9 PA10 PA11 PA12.

PA13 PA14 PA15 PA19 PA20 PA21 PA22 PA23 PA24.

/PRINT UNIVARIATE INITIAL KMO EXTRACTION ROTATION.

/FORMAT SORT BLANK(0.30)

/CRITERIA FACTORS(4) ITERATE(25)

/EXTRACTION PAF.

/CRITERIA ITERATE(25)

/ROTATION PROMAX.

/METHOD = CORRELATION.

# reliability analysis #

RELIABILITY.

/VARIABLES=PA11 PA13 PA9 PA10 PA12 PA15 PA14.

/SCALE('ALL VARIABLES') ALL.

/MODEL = ALPHA.

RELIABILITY.

/VARIABLES=PA19 PA20 PA21.

/SCALE('ALL VARIABLES') ALL.

/MODEL = ALPHA.

RELIABILITY.

/VARIABLES=PA3 PA2 PA_PI1 PA7 PA4 PA6 PA5.

/SCALE('ALL VARIABLES') ALL.

/MODEL = ALPHA.

RELIABILITY.

/VARIABLES=PA23 PA24 PA22.

/SCALE('ALL VARIABLES') ALL.

/MODEL = ALPHA.

#CFA using the second dataset (data3) in lavaan package in R#

# applying the necessary packages #

CFA < -c("foreign", "semTools", "semPlot", "lavaan")

lapply(CFA, require, character.only = TRUE)

# calling for the dataset #

data = read.spss(file.choose, to.data.frame = TRUE, value.data.labels = TRUE)

View(data)

attach(data)

# CFA syntax #

models < -list

fits < -list

# model 1 #

models$PA1<−'f1 = ∼ PA11+PA13+PA9+PA10+PA12+PA15+PA14

f2 = ∼ PA19+PA20+PA21

f3 = ∼ PA3+PA2+PA_PI1+PA7+PA4+PA6+PA5

f4 = ∼ PA23+PA24+PA22'

fits$PA1<-cfa(models$PA1, data = data)

summary(fits$PA1, fit.measures = TRUE, standardized = TRUE)

modificationindices(fits$PA1)

# model 2 #

models$PA2<−'f1 = ∼ PA11+PA13+PA9+PA10+PA12+PA15+PA14

f2 = ∼ PA19+PA20+PA21

f3 = ∼ PA3+PA2+PA_PI1+PA7+PA4+PA6+PA5

f4 = ∼ PA23+PA24+PA22.

PA11 ∼∼ PA10.

PA9 ∼∼ PA12.

PA10 ∼∼ PA5.

PA10 ∼∼ PA_PI1'

fits$PA2<-cfa(models$PA2, data = data)

summary(fits$PA2, fit.measures = TRUE, standardized = TRUE)

modificationindices(fits$PA2)

# model 3 #

models$PA3<−'f1 = ∼ PA11+PA13+PA9+PA10+PA12+PA15+PA14

f2 = ∼ PA19+PA20+PA21

f3 = ∼ PA3+PA2+PA_PI1+PA7+PA4+PA6+PA5

f4 = ∼ PA23+PA24+PA22.

PA10 ∼∼ PA5.

PA2 ∼∼ PA_PI1.

PA21 ∼∼ PA24.

PA9 ∼∼ PA12.

PA9 ∼∼ PA15.

PA11 ∼∼ PA10.

PA12 ∼∼ PA3.

PA9 ∼∼ PA10.

PA9 ∼∼ PA4.

PA10 ∼∼ PA4'

fits$PA3<-cfa(models$PA3, data = data)

summary(fits$PA3, fit.measures = TRUE, standardized = TRUE)

# reliability syntax for the CFA is the same as the EFA, using model 3 of the CFA dataset #
